# Function Prediction of Peptide Toxins with Sequence-Based Multi-Tasking PU Learning Method

**DOI:** 10.3390/toxins14110811

**Published:** 2022-11-21

**Authors:** Yanyan Chu, Huanhuan Zhang, Lei Zhang

**Affiliations:** 1 School of Medicine and Pharmacy, Ocean University of China, Qingdao 266003, China; 2 Pilot National Laboratory for Marine Science and Technology (Qingdao), Qingdao 266200, China; 3 Marine Biomedical Research Institute of Qingdao, Ocean University of China, Qingdao 266003, China

**Keywords:** peptide toxin, active peptide, function prediction, PU learning, sequence-based

## Abstract

Peptide toxins generally have extreme pharmacological activities and provide a rich source for the discovery of drug leads. However, determining the optimal activity of a new peptide can be a long and expensive process. In this study, peptide toxins were retrieved from Uniprot; three positive-unlabeled (PU) learning schemes, adaptive basis classifier, two-step method, and PU bagging were adopted to develop models for predicting the biological function of new peptide toxins. All three schemes were embedded with 14 machine learning classifiers. The prediction results of the adaptive base classifier and the two-step method were highly consistent. The models with top comprehensive performances were further optimized by feature selection and hyperparameter tuning, and the models were validated by making predictions for 61 three-finger toxins or the external HemoPI dataset. Biological functions that can be identified by these models include cardiotoxicity, vasoactivity, lipid binding, hemolysis, neurotoxicity, postsynaptic neurotoxicity, hypotension, and cytolysis, with relatively weak predictions for hemostasis and presynaptic neurotoxicity. These models are discovery-prediction tools for active peptide toxins and are expected to accelerate the development of peptide toxins as drugs.

## 1. Introduction

Peptide toxins are mostly found in the venoms of toxic organisms, such as cone snails, sea anemones, snakes, spiders, scorpions, and centipedes [1]. Venoms provide a wide variety of peptide toxins, and even within intraspecific venom, the composition may not overlap [2,3,4]. Once their biological function is known, peptide toxins can be used in different research areas; for example, vasoactive peptides can be used as biomarkers to predict cancer risk [5], and neurotoxins may have potential applications in the treatment of certain neurological disorders, e.g., presynaptic neurotoxins have been used to treat migraine headache and cerebral palsy [6].

Peptide toxins are specific in their amino acid composition. They usually contain several intramolecular disulfide bonds, which confer chemical, thermal, and biological stability. To facilitate prey capture and help defend against potential threats, toxins often interfere with the victim’s core physiological systems and signaling processes with exceptional specificity and high potency [2,7]. Thus, despite the obvious health concerns of being exposed to toxins, peptide toxins provide a rich source of novel bioactive molecules.

Peptide toxins have abundant biological activities, mainly by binding to sodium (Na), potassium (K) or calcium (Ca) ion channels and receptors in the nervous system with high selectivity. Many peptide toxins are used as sensitive pharmacological tools to unravel disease pathways and to tease out the functional importance of different subtypes of ion channels [7,8,9,10,11,12,13]. Due to the binding specificity for particular molecular targets and the critical roles of ion channels, peptide toxins have the potential to be applied as lead structures for the development of drugs to treat a wide variety of diseases. Seven toxin-derived products are approved by FDA [2], including angiotensin converting enzyme (ACE) inhibitor captopril from the venom of *Bothrops jararaca* for the treatment of hypertension [14], antiplatelet drug eptifibatide for the treatment of acute coronary syndrome [15,16], ziconotide designed from ω-conotoxin MVIIA for pain treatment [17,18], and a Gila-monster toxin derivative for treatment of type II diabetes (Byetta^®^, AstraZeneca, London, UK) [13]. Many additional toxins are being explored and developed into drugs for the treatment of conditions such as cardiovascular disorders, chronic pain, inflammation, hypertension, thrombosis, cancer and neurological disorders [13,19,20,21,22,23]. 

Considerable effort has been devoted to the study of the biological activity of peptide toxins. However, a large number of natural peptide toxins remain unexplored. The traditional functional studies of peptide toxins are time-consuming and costly. Machine learning and deep learning technologies can analyze potential patterns and rapidly make predictions from large amounts of biological data. Currently, these technologies have been successfully applied to the discovery of bioactive peptides such as antimicrobial peptides (AMPs) [24,25], anticancer peptides (ACPs) [26,27] and anti-inflammatory peptides (AIPs) [28], most of which are synthetic peptides. The composition, physicochemical properties and physiological activity of synthetic and peptide toxins are quite different. For peptide toxins, Yang et al. [29] published a model to distinguish between presynaptic and postsynaptic neurotoxins with true positive ratio at 0.91~1. ClanTox can be used to classify short animal toxin and toxin-like protein [30]. However, the lack of tools for function prediction of peptide toxins hinders drug discoveries from these toxins. 

In this study, we developed a tool to predict the physiological characteristics of peptide toxins by using sequence-based multi-task positive-unlabeled (PU) learning methods. A total of 9294 peptide toxins from Uniprot database [31] were used to establish and validate the models. Among the toxins, there are 61 three-finger toxins, which were extracted and used as external validation. For each model, the peptides were binary categorized by their activities. Since for a specific activity category the number of active peptides was much less than the number of peptides with undetermined activity, we adopted three PU learning schemes [32] to build reliable models for each activity category. This proved that the adaptive basis classifier and two-step method were suitable for this study, while the bagging method was not comparable with these two schemes. We compared 14 classifiers for each scheme, and eight evaluation metrics were used for each model. After feature selection, the optimal parameter combinations for the outstanding models were further optimized using stratified tenfold cross-validation. Finally, one model from the adaptive base classifier and the two-step approach was selected for each function prediction. The models were validated by making predictions on external HemoPI datasets and three-finger toxins. These models can be combined and used to screen active peptides and guide bioactivity studies of new peptide toxins.

## 2. Results and Discussions

### 2.1. Data Distribution

A total of 10,205 peptide toxins, containing 10~100 amino acids, were retrieved from Uniprot database. After removal of the signal peptide fragment, the duplicate sequence was discarded, leaving 9294 peptides. Fifty-six physicochemical properties (47 peptide descriptors and 9 global descriptors) were calculated for each peptide based on its mature peptide sequence, and these physicochemical properties were normalized and used for model construction and prediction. 

To validate the final models, 61 three-finger toxin peptides, which performed a wide variety of functions, including cytolysis, cardiotoxicity, neurotoxicity, and postsynaptic neurotoxicity, were extracted as the external validation set. For each specific physiological function, the remaining 9233 peptide toxins were binary classified according to whether they were active or not. Among these peptides, 7386 peptides (80% of 9233) were randomly selected for model training and the remaining 1847 peptides for model testing. 

**Figure 1:**
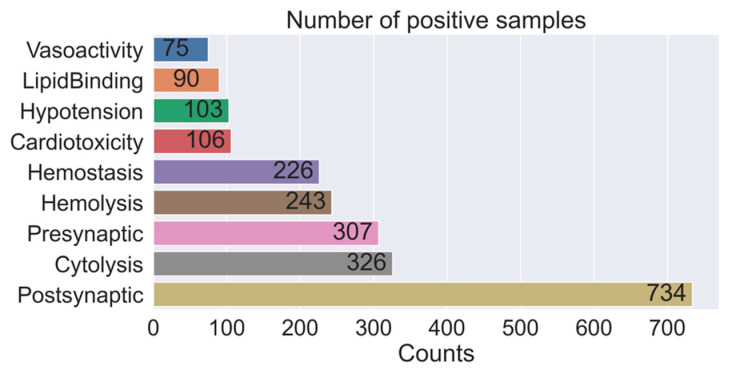
Statistics on the number of positive samples for each activity category.

The peptide toxins in the dataset mainly included 3302 neurotoxins, 106 cardiotoxins, 326 cytolytic peptides, 243 hemolytic peptides, 226 hemostatic peptides, 103 hypotensive agents, 90 lipid-binding peptides, and 75 vasoactive peptides (Figure 1). Among the neurotoxins, there were 734 postsynaptic neurotoxins and 307 presynaptic neurotoxins. Some peptide toxins have been widely studied and contain multiple physiological activities. However, many more of these toxins have undetermined biological activities, i.e., they may be active or inactive. 

The three HemoPI datasets [33], HemoPI1-3, consist of experimentally validated hemolytic peptides that are toxin-independent. These datasets had been preprocessed for machine learning [25], and active and inactive peptides were clearly labeled. HemoPI1 contains 552 hemolytic peptides and 552 random non-hemolytic peptides. HemoPI2 contains 552 highly active hemolytic peptides and 462 inactive hemolytic peptides. HemoPI3 contains 885 highly hemolytic and 738 poorly hemolytic peptides. These three datasets were used to validate and improve the performance of the hemolysis model.

### 2.2. Algorithm Selection

Although conventional supervised binary classification algorithms have been widely used in biological and pharmaceutical fields [33,34], they require fully labeled classes of data (positive and negative samples) to train a classification model. In the Uniprot dataset, the inactive peptides were not determined, so conventional schemes are not appropriate for this study. The PU learning schemes enable the classifier to learn directly from a limited number of positive samples and a large number of unlabeled samples, and have been successfully used for biological sequence classification [35]. Therefore, three different PU learning schemes, adapting base classifier [36], two-step [37] method, and PU bagging [38], were adopted in this study. Since no single machine-learning method or class of methods always performs best [39], 14 classification algorithms were compared to efficiently identify the positive sample: logistic regression (LR) [40], linear (LDA) and quadratic (QDA) discriminant analysis [41], support vector machine classifier (SVC) with 3 kernels radial basis function (SVC_rbf), polynomial (SVC_poly) and sigmoid (SVC_sig) [42], K-neighbors classifier (KNN) [43], Gaussian naïve bayes (GNB) [44], decision trees classifier (DTC) [45], neural network multilayer perceptron (MLP) [46], random forest classifier (RF) [47], AdaBoost classifier (Ada) [48], gradient boosting classifier (GBC) [49] and LightGBM classifier (LGBM) [50].

The performance of each model was evaluated by true positive ratio (TPR), balanced accuracy (bAcc), weighted precision (P), weighted recall (R), Matthew’s correlation coefficient (MCC), F1 score (F1), area under the curve receiver operating characteristic (AUC-ROC) and area under the precision and recall curve (AUPR). MCC and F1 are both popular metrics for imbalanced class problems. F1 is defined as the harmonic mean of precision and recall, which can be interpreted as the weighted average of precision and recall. AUPR, another performance metric for imbalanced data, places more emphasis on finding positive samples. All evaluation metrics except MCC range from 0 to 1, and MCC is in the range of −1 to 1. For each evaluation metric, the higher the value, the better the model performance. A value of 0 for MCC and 0.5 for the other metrics indicates an average random prediction. 

### 2.3. Adapting Base Classifier 

The adapting base classifier adapts the base classifiers to focus on estimating and correcting the proportion of positive and negative samples in the unlabeled dataset [32]. Based on the ‘selected completely at random’ assumption [36], the ratio of positive and negative samples in the unlabeled dataset is estimated by dividing the score by a constant correction factor, which is usually obtained from a separate validation set [32].

**Figure 2:**
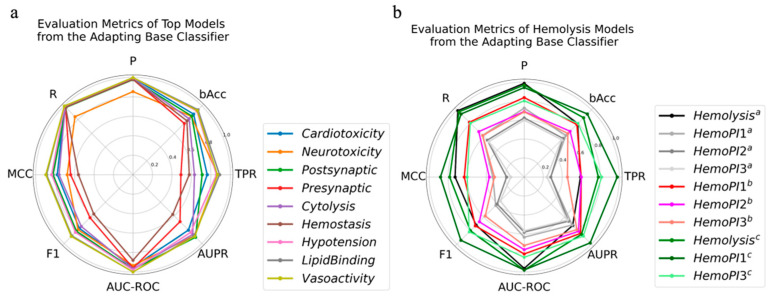
The optimal models selected from the adapting base classifier and their evaluation metrics on test/validation set. (**a**) The optimal models for the prediction of cardiotoxicity, neurotoxicity, postsynaptic neurotoxicity, presynaptic neurotoxicity, cytolysis, hemostasis, hypotension, lipid binding, and vasoactivity. (**b**) The evaluation metrics of the optimal hemolysis model on test set and the HemoPI datasets. The superscript a indicates that the model is built on the Uniprot dataset, the superscript b indicates that outliers are removed from the datasets, and the superscript c indicates that the model is built on the combination of the Uniprot and HemoPI2 datasets.

Based on this strategy, an optimal model was selected for each specific physiological function prediction by comprehensively considering the eight evaluation metrics (Figure 2a and Table S1). Prediction models for cardiotoxicity, neurotoxicity, postsynaptic neurotoxicity, hemostasis, hypertension, and vasoactivity were obtained by training the KNN classifier, for presynaptic neurotoxicity, cytolysis, and hemolysis by training the LGBM classifier, and for lipid binding by training the SVC_poly classifier. The evaluation metrics of hypotension, lipid binding, and vasoactivity prediction models are all above 0.8, implying that they are reliable. Most of the evaluation metrics of other models were close to or above 0.8. The lowest evaluation metrics were from the hemostasis and presynaptic neurotoxicity prediction models with TPR, MCC, F1, and AUPR values around 0.6.

The R, P, and AUC-ROC values of the best hemolysis prediction model were close to 1, the bAcc value was 0.8, and the MCC, F1, and AUPR values fell in the range of 0.6 to 0.8 (Figure 2b). The model was used to make predictions for the entire data set of the three HemoPI. The results indicated that our hemolysis prediction model performed weakly, with P, R, AUC-ROC, AUPR, and bAcc around 0.6 (slightly greater than 0.5), with MCC slightly greater than 0.

**Figure 3:**
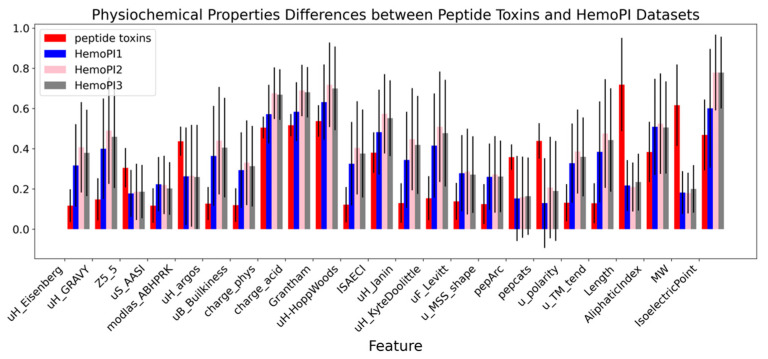
The mean values of 24 physiochemical properties with significant differences between peptide toxins from Uniprot and those from HemoPI1, HemoPI2 and HemoPI3. The X-axis labels the physicochemical properties. A standard error bar was added onto the top of each bar.

The physiochemical properties of the Uniprot dataset and the three HemoPI datasets after normalization were compared to better understand the model and to find out the reasons for its weak performance. The mean and standard errors for each property were calculated. The distribution of physiochemical properties was similar for the three HemoPI datasets (Figure 3). However, there were significant differences in the range of values for 24 out of the 56 properties between the peptides from Uniprot and those from HemoPI datasets. Notably, there was little overlap in the range of values for a variety of properties, including peptide size-related properties such as length, MW, and uB_Builkiness, hydrophobicity-related properties such as uH_Eisenberg, uH_GRAVY, uH_argos, uH_HoppWoods, uH_Janin, and uH_KyteDoolittle, hydrophilicity-related properties such as charge_acid, and pharmacophoric features such as pepArc and pepcats. The meaning of the properties was listed in List S1. These significant differences resulted in weak performance of the hemolysis peptide prediction model on HemoPI peptides.

After removing outliers from the three HemoPI datasets using the average KNN of PyOD [48], the lines on evaluation metrics were located outside any of the original evaluation metric lines, which indicated that the predictive power of the hemolysis prediction model was significantly enhanced (Figure 2b). From the radar diagram, it is easy to see that the evaluation metric lines for HemoPI1, HemoPI2, and HemoPI3 are from the outside in order, which means that the model has the best prediction for the HemoPI1 dataset, followed by HemoPI2 and HemoPI3. The model predictions on HemoPI1 were evaluated by R, P, bAcc, AUC-ROC, and AUPR of about 0.8, F1 of about 0.7, and MCC and TPR of about 0.6. 

To extend the application of the hemolysis prediction model and to further validate the methods, HemoPI2 was involved in the retraining of the hemolysis LGBM model. HemoPI1 and HemoPI3 datasets were used to validate the new models. The results showed that the performance of the new model was further improved (Figure 2b). Almost all of the evaluation metrics on the test set, HemoPI1 and HemoPI3 datasets were above 0.8, except for MCC of HemoPI3 around 0.6. The evaluation metrics for HemoPI1 and HemoPI3 were P: 0.94 and 0.80, bAcc: 0.94 and 0.80, MCC: 0.88 and 0.60, and AUC-ROC: 0.98 and 0.85, respectively. Plisson et al. [25] published two independent HemoPI1 and HemoPI3 models, and the corresponding evaluation metrics for HemoPI1 and HemoPI3 were P: 0.94 and 0.75, Acc: 0.96 and 0.79, MCC: 0.91 and 0.57, and AUC-ROC: 0.96 and 0.78. Thus, our hemolysis prediction model was reliable and robust, and it performed comparably well to published ones. Overall, the adapting base classifier was suitable for this system.

### 2.4. Two-Step Method

The two-step method adopts two independent algorithms in turn; the first one identifies reliable negatives from the unlabeled datasets based on the likelihood, and the second one is trained to make predictions [32]. In this study, 14 classifiers were iteratively used to identify reliable negative samples from unlabeled data and to train the classifier. After training the classifiers, similarly, an optimal model was selected for each specific physiological function prediction. It turned out that the KNN was a suitable classifier for each model establishment to identify reliable negatives in the first step. The optimal models for cardiotoxicity, neurotoxicity, postsynaptic neurotoxicity, hemostasis, hypotension, and vasoactivity prediction were developed by training the KNN classifier, for presynaptic neurotoxicity, cytolysis, and hemolysis prediction by training the LGBM classifier, and for lipid binding prediction by training the SVC_poly classifier.

**Figure 4:**
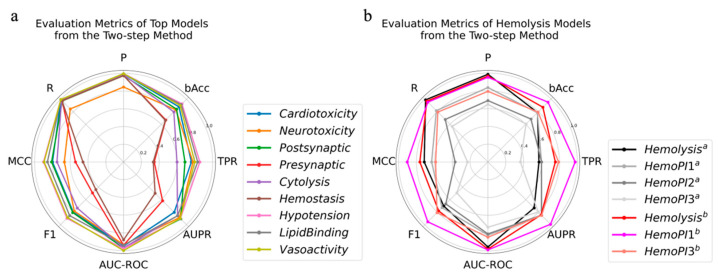
The evaluation metrics of the optimal models based on the two-step method. (**a**) The evaluation metrics of the optimal models on the test set. (**b**) The evaluation metrics of optimal hemolysis model on the test set and HemoPI datasets with outliers removed, where superscript a indicates that the model is built on the Uniprot dataset, and superscript b indicates that the model is built on the combination of the Uniprot and HemoPI2 datasets.

Prediction models for cardiotoxicity, hypotension, lipid binding, and vasoactivity were robust on the test set, with each evaluation metric near or above 0.8 (Figure 4a). The lowest evaluation metric of the neurotoxicity prediction model, MCC, was above 0.6, indicating that it was able to distinguish between active and inactive peptides in the test set. The evaluation metrics of the prediction models for cytolysis and postsynaptic neurotoxicity were greater than 0.8, except for TPR values at 0.6~0.7, which meant that 60~70% of active peptides were found by these two models. The presynaptic neurotoxicity and hemostasis prediction models, with MCC of about 0.5 and bAcc of 0.6–0.7, had a weak predictive power, and their TPR values were less than 0.4, indicating that most of the active peptides were not identified.

For the hemolysis prediction model, all evaluation metrics were close to those of the model based on the adapting base classifier, with MCC, F1, AUPR and bAcc ranging from 0.6 to 0.8 (Figure 4b). After removing outliers, these two models had comparable predictive power for the HemoPI1 and HemoPI2 datasets, and they both had worse predictive power for HemoPI3 (Figure 2b and Figure 4b). On the HemoPI3 dataset, the model based on the two-step method identified fewer positives (36%) compared to the 45% positives identified by the model based on the adaptive base classifier. To improve the performance of the model, the hemolysis model was re-trained after adding HemoPI2 into the training set. The optimal model was still obtained by training the LGBM classifier. The performance of the new model was improved, especially the TPR, by almost 20% on the test set. The model predicted HemoPI1 with each evaluation metric above 0.9. For the HemoPI3 dataset, all evaluation metrics were about 0.8, except for MCC at 0.57, which verified the reliability of the model.

### 2.5. PU Bagging 

In the PU bagging [32], unlabeled samples are grouped into several smaller subsamples using bootstrap aggregation. The subsamples were treated as negatives and used for building a model along with the positive samples. After a certain number of iterations, the average score of each sample was calculated, and the sample was predicted to be positive if the value reached a threshold. In this scheme, we performed 128 resamplings and model fittings for each model on each of the 14 classifiers (Table S4). None of the models performed as well as the models from the other two schemes. 

### 2.6. Feature Selection

To optimize the models, the informative and non-redundant features were selected based on the Pearson product-moment correlation coefficients. We set the coefficient threshold to 0.02, i.e., features with coefficient less than 0.02 were considered irrelevant to the prediction target and discarded. Each model was re-trained using the refined features. In general, no significant changes in model performance were observed before and after feature selection for either the adaptive base classifier (Tables S1 and S3) based models or the two-step method based models (Tables S5 and S6). Nevertheless, feature selection is recommended because it saves computational resources. This result, in turn, confirmed that features with coefficient less than 0.02 were irrelevant to the prediction target and could be discarded. Although the performance of the model remained unchanged, feature selection is recommended because it saves computational resources. 

To further evaluate the performance of the models, we compared the prediction results of the corresponding optimal models from the two schemes. The prediction results of the models for the prediction of cytolysis, lipid binding, vasoactivity, and hypotension were highly consistent. Among the peptides, unlabeled helokinestatin (Uniprot ID: P86446) was unanimously recognized as a hypotensive agent, which was consistent with previous reports [51,52]. 

### 2.7. Hyperparameter Tuning

To obtain an optimal parameter combination, GridSearchCV was run to perform a grid search with stratified tenfold cross-validation. Stratified tenfold cross-validation is a variant of tenfold cross-validation where the folds are stratified, which means it splits the dataset in a way that maintains the same class distribution in each subset. Feature selection as mentioned above was performed in the parametric grid search process. In Table 1, we gathered the best prediction models based on the adapting base classifier, their optimal parameter combinations, and their evaluation metrics (Table 1). The predictive models for cardiotoxicity, postsynaptic neurotoxicity, cytolysis, vasoactivity, and hypotension performed well, with all evaluation metrics above 0.8, followed by the neurotoxicity, lipid binding, and hemolysis prediction models with P between 0.74 and 0.77. The hemolysis model was able to identify 99% of the hemolytic peptides in the HemoPI1 dataset and 87% in the HemoPI3 dataset. The other two prediction models for hemostasis and presynaptic neurotoxicity had weak predictive power with TPRs of 0.60 and 0.70, respectively. 

Similarly, the optimal parameter combinations of the models based on the two-step method were explored. Only the informative features, with Pearson product-moment correlation coefficients above 0.02, were used to train the models. The first step of each model construction used the KNN classifier to identify reliable negatives. The optimal model and parameter combination adopted in the second step are shown in Table 2. The prediction models for cardiotoxicity, postsynaptic neurotoxicity, cytolysis, vasoactivity, hypotension, and lipid binding performed well on the test set, with all evaluation metrics above 0.8, followed by neurotoxicity, hemolysis, and cytolysis, which were able to identify 85%, 82%, and 77% of the corresponding active peptides, respectively. The presynaptic neurotoxicity and hemostasis prediction models performed worse than the corresponding models based on the adaptive base classifier, with TPRs of 0.51 and 0.36 and AUPRs of 0.64 and 0.55, respectively. The hemolysis model made accurate and precise predictions on HemoPI1 and HemoPI3, and identified 99% of the hemolytic peptides in the HemoPI1 dataset with a P value of 0.89 and 85% in the HemoPI3 dataset with a P value of 0.79.

### 2.8. Model Validation

To further assess the quality of the models, these models were used to make predictions for 61 three-finger toxins (Table 3, Tables S9 and S10). These toxins include 7 cytolytic toxins, 7 cardiotoxins, 31 neurotoxins (30 of which are presynaptic neurotoxins), 2 hemolytic toxins, and 21 peptides with no reported activity. Six of the cytolytic toxins are also cardiotoxins and one is a neurotoxin. The prediction results showed that the adaptive base classifier-based models identified 100% of the cytolytic toxins, cardiotoxins and neurotoxins, and identified 22 of the 30 postsynaptic neurotoxins. In addition, the models identified several active peptides from unlabeled peptides, including 3 cytolytic toxins, 19 neurotoxins, 7 postsynaptic neurotoxins, and 2 hemostatic agents. The models based on the two-step method identified 6 cytolytic toxins, 7 cardiotoxins, 30 neurotoxins, and 22 postsynaptic neurotoxins. Almost all of the unlabeled peptides were identified as inactive. The models also identified several active peptides from unlabeled peptides, including a cytolytic toxins, 18 neurotoxins, 7 postsynaptic toxins, a hypotensive agent, and a hemostatic agent. Among these peptides, the unlabeled toxin MicTx3 was predicted to be a neurotoxin by both models, which is consistent with its reported inhibition of nicotinic acetylcholine receptor (nAChR) α7 activity [53], which is expressed in the brain, both presynaptically and postsynaptically. Another unlabeled three-finger toxin MALT0044C, predicted to be a neurotoxin by the adapting base classifier-based model and as a postsynaptic neurotoxin by both models, has been reported to have neurotoxic activity [54,55]. Overall, the performance of the adaptive base classifier-based models and the two-step-based models is comparable. These models can be used in combination to further increase confidence in the prediction results.

## 3. Conclusions

Peptide toxins generally have extreme pharmacological activities, providing a rich source for the discovery of potential pharmaceuticals. Machine learning and deep learning technologies have provided time-saving and cost-effective solutions for function prediction of peptide toxins. In this study, we developed multiple models to predict the functions of peptide toxins based on peptide physicochemical property descriptors, which were calculated based on primary sequence. These models were chosen by comparing the performance of three PU learning schemes, each of which was sequentially embedded with 14 machine learning classifiers. It turned out that both the adapting base classifier and the two-step method are applicable to the function predictions of peptide toxins. The initial hemolysis model from adapting base classifier was comparable to the published models in an inlier peptide dataset. To expand the applicable scope of the model, HemoPI2 and the Uniprot dataset were combined to re-train the model using both adapting base classifier and two-step schemes, and the new models outperformed existing models. As with the hemolysis models, the applicability of our models is limited by the distribution of the data they fitted, which came from peptide toxins with less than 100 residues. Therefore, we strongly recommend implementing outlier exclusion methods, such as average KNN, to remove outliers before making predictions. Finally, reliable predictive models for cardiotoxicity, cytolysis, hypotension, lipid binding, neurotoxicity, postsynaptic neurotoxicity, vasoactivity, and hemolysis were developed. The models for the prediction of hemostasis and presynaptic neurotoxicity possessed weak predictive power. Model validation by an external validation set showed that our models made robust predictions for 61 three-finger toxins. Two unlabeled peptides were predicted to be neurotoxins and their activity was verified. Our models will be used to screen for bioactive peptides, and to guide the bioactivity studies of new peptide toxins, thereby accelerating the development of peptide toxins as drugs.

## 4. Methods

**Figure 5:**
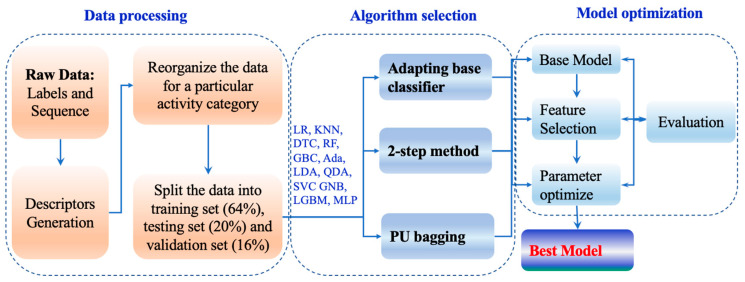
The workflow of this study.

**General workflow.** The general workflow is shown in Figure 5. Firstly, peptide toxins and hemolytic peptide datasets were collected from Uniprot database [31] and previous research [25], respectively. Then, the data from Uniprot were cleaned and preprocessed. Peptide descriptors were generated for each peptide based on its primary sequences. After normalization, 61 three-finger toxins were isolated as an external validation set, and the remaining data were divided into a training set and a test set. Fourteen algorithms were then embedded in three schemes for PU learning [32]. The performance of the models was further improved by feature selection and optimizing parameters in turn. The hemolytic peptide datasets were used to evaluate and improve the performance of the hemolysis model. 

All computational studies were developed in Jupyter notebooks using various Python modules for data preprocessing and analysis, calculating peptide descriptors and machine learning algorithms, which will be described in detail.

**Datasets.** The sequence and related information of a total of 10,205 peptide toxins, containing 10~100 amino acids, were retrieved from Uniprot database [31]. Each sequence was first cleaned by removing peptides with non-standard residues and cleaving the signal peptide fragment. After removing duplicate sequences, 9294 samples were kept. Among them, 61 three-finger toxins were isolated as the external validation set. The remaining 9233 samples were randomly stratified into two datasets, a training set, accounting for 80%, for constructing the model and a test set, accounting for 20%, for evaluating the model. 

**HemoPI datasets.** Three HemoPI datasets (HemoPI1, HemoPI2 and HemoPI3) containing experimentally validated hemolytic peptides were downloaded from Github, published by Fabien Plisson [25]. The datasets had been preprocessed for machine learning [25]. The datasets were used to validate and improve the performance of the hemolysis model. HemoPI2 was also used in training some models.

**Physicochemical properties.** Based on the peptide primary sequence, 56 physicochemical properties (47 peptide and 9 global descriptors) for each peptide were calculated using modlamp 4.3.0 [56]. The calculated properties were transformed to a range of 0~1 using MinMaxScaler of sklearn preprocessing module [57]. The data were then used as the input for model construction.

**PU schemes.** Three PU schemes were used to develop the best models, adapting base classifier [36], two-step method [37] and PU bagging [38]. For each model, the data were split into a training set (80%, 7386 samples) and a validation set (20%, 1847 samples). The positive samples of the training set were labeled as 1, and other unlabeled samples were labeled as −1. For each scheme, 14 machine learning algorithms were compared: LR, LDA, QDA, SVC_rbf, SVC_poly, SVC_sig, KNN, GNB, DTC, MLP, RF, Ada, GBC and LGBM. 

The adapting base classifier focuses on estimating the proportion of positive and negative samples in the unlabeled dataset. Based on the “selected completely at random” assumption [36], the ratio of positive and negative samples in the unlabeled dataset was estimated by dividing the score by a constant correction factor Px (y = 1), which was calculated by Equation (1).

Px (y = 1) = (Px (S = 1))/(Px (S = 1/y = 1)) (1)

where Px (S = 1) is the probability of sample x being labeled as positive by a base classifier. Px (S = 1/y = 1) is the constant probability that a positive example is labeled. In our model, this was calculated as the probability mean of 20% of the positive samples from the training set. The classification results were obtained by an average of 24 iterations.

In the two-step scheme [37,58], the first step is to identify reliable negative samples from unlabeled data for the first step. The second step is to train the classifier by supervised learning algorithms using the filtered reliable negative and positive samples. We applied 14 machine learning algorithms independently for each step. The second step was iterated 10 times in order to accurately screen out more negative samples. 

PU bagging is a popular bootstrap aggregating approach to increase classifier performance [38]. To obtain the best model, we performed 128 resamplings and model fittings for each model on each algorithm. 

**Performance evaluation.** To measure the quality of the classifiers, the following evaluation metrics were applied: true positive ratio (Equation (2)), balanced accuracy (bAcc.) (Equation (3)), weighted precision (P) (Equation (4)), weighted recall (R) (Equation (5)), Matthews correlation coefficient (MCC) (Equation (6)), F1 score (F1) (Equation (7)), area under the curve receiver operating characteristic (AUC-ROC) and area under the precision and recall curve (AUPR).

TPR = TP/(TP + FN) (2)

bAcc = 1/2 (TP/(TP + FN) + TN/(FP + TN)) (3)

P = (P/(P + N))(TP/(TP + FP)) + (N/(P + N))(TN/(TN + FN)) (4)

R = (P/(P + N))(TP/(TP + FN)) + (N/(P + N))(TN/(TN + FP)) (5)

MCC = (TP × TN − FP × FN)/((TP + FP)(TP + FN)(TN + FP)(TN + FN))^1/2^ (6)

F1 = 2PR/(P + R) (7)

where TP, TN, FP, and FN are the number of true positives, true negatives, false positives and false negatives, respectively; P and N are the number of positives and negatives.

**Feature elimination.** The Pearson product-moment correlation coefficient (Equation (8)) was applied to extract features. The correlation of each feature against a particular label was calculated. Features with correlation values greater than 0.02 were considered informative and non-redundant, and other features were deleted.

Rij = Cij/(Cii·Cjj)^1/2^ (8)

where R is the correlation coefficient matrix, C is the covariance matrix, and i and j are two features. 

**Hyperparameter tuning.** A limited number of specific hyperparameters were predefined. The hyperparameters are tuned automatically through a grid search with cross-validation (GridSearchCV) of scikit-learn’s model_selection package [57]. Stratified tenfold cross-validation was used. Through the cycle of hyperparameters, the optimal parameter combination was obtained according to the AUPR value of the model.

## Figures and Tables

**Table 1:** Information on the top performing models from adapting base classifier after hyperparameters tuning.

Activity	Model	Parameters	TPR	AUPR	R	bAcc	F1	P	MCC	AUC-ROC
Cardiotoxicity	KNN	{‘n_neighbors’: 5, ‘p’: 1, ‘weights’: ‘distance’}	0.81	0.83	0.81	0.90	0.83	0.85	0.83	0.95
Neurotoxicity	LGBM	{‘max_depth’: 8, ‘num_leaves’: 40}	0.89	0.90	0.86	0.81	0.74	0.85	0.70	0.94
Postsynaptic	LGBM	{‘max_depth’: 8, ‘num_leaves’: 40}	0.91	0.82	0.91	0.86	0.90	0.98	0.85	0.97
Presynaptic	LGBM	{‘max_depth’: 8, ‘num_leaves’: 40}	0.70	0.66	0.71	0.85	0.65	0.61	0.64	0.95
Cytolysis	LGBM	{‘max_depth’: 7, ‘num_leaves’: 30}	0.80	0.87	0.80	0.90	0.85	0.90	0.84	0.97
Hemostasis	KNN	{‘n_neighbors’: 3, ‘p’: 4, ‘weights’: ‘distance’}	0.60	0.58	0.60	0.80	0.61	0.63	0.60	0.88
Vasoactivity	KNN	{‘n_neighbors’: 5, ‘p’: 2, ‘weights’: ‘distance’}	0.93	0.99	0.93	0.97	0.93	0.93	0.93	1.00
Hypotension	KNN	{‘n_neighbors’: 6, ‘p’: 1, ‘weights’: ‘distance’}	0.86	0.89	0.86	0.93	0.88	0.90	0.88	0.97
Lipid binding	LGBM	{‘max_depth’: 8, ‘num_leaves’: 20}	0.94	0.87	0.94	0.97	0.85	0.77	0.85	0.99
Hemolysis ^a^	LGBM	{‘max_depth’: 7, ‘num_leaves’: 20}	0.82	0.84	0.82	0.90	0.78	0.74	0.76	0.98
HemoPI1 ^b^			0.99	0.96	0.99	0.85	0.87	0.78	0.73	0.95
HemoPI3 ^b^			0.87	0.88	0.87	0.78	0.82	0.77	0.57	0.84

Note: ^a^ The model was trained on the data from Uniprot and HemoPI2; ^b^ The external validation dataset was not used to train the model, but only to validate the hemolysis model.

**Table 2:** Information on the two-step models after hyperparameters tuning.

Activity	Model	Parameters	TPR	AUPR	R	bAcc	F1	P	MCC	AUC-ROC
Cardiotoxicity	KNN	{‘n_neighbors’: 2, ‘weights’: ‘uniform’}	0.81	0.87	1.00	0.90	0.85	1.00	0.85	0.95
Neurotoxicity	LGBM	{‘max_depth’: 8, ‘num_leaves’: 40}	0.85	0.90	0.86	0.86	0.81	0.86	0.70	0.94
Postsynaptic	LGBM	{‘max_depth’: 8, ‘num_leaves’: 40}	0.80	0.91	0.98	0.90	0.86	0.98	0.86	0.97
Presynaptic	LGBM	{‘max_depth’: 6, ‘num_leaves’: 40}	0.51	0.64	0.98	0.75	0.57	0.97	0.56	0.94
Cytolysis	LGBM	{‘max_depth’: 6, ‘num_leaves’: 30}	0.77	0.87	0.99	0.88	0.81	0.99	0.80	0.97
Hemostasis	KNN	{‘n_neighbors’: 4, ‘p’: 2, ‘weights’: ‘distance’}	0.36	0.55	0.98	0.68	0.48	0.98	0.50	0.87
Vasoactivity	KNN	{‘n_neighbors’: 8, ‘p’: 2, ‘weights’: ‘distance’}	0.87	1.00	1.00	0.93	0.93	1.00	0.93	1.00
Hypotension	KNN	{‘n_neighbors’: 1, ‘weights’: ‘uniform’}	0.86	0.86	1.00	0.93	0.86	1.00	0.86	0.93
Lipid binding	SVM	{‘C’: 100.0, ‘gamma’: 1.0, ‘kernel’: ‘rbf’}	0.94	0.92	1.00	0.97	0.90	1.00	0.90	0.99
Hemolysis ^a^	LGBM	{‘max_depth’: 8, ‘num_leaves’: 40}	0.82	0.85	0.96	0.90	0.78	0.97	0.76	0.98
HemoPI1 ^b^			0.99	0.98	0.87	0.87	0.88	0.89	0.75	0.98
HemoPI3 ^b^			0.85	0.85	0.79	0.78	0.81	0.79	0.57	0.85

Note: ^a^ The model was trained on the data from Uniprot and HemoPI2; ^b^ The external validation data were not used to train the model, but only to validate the hemolysis model.

**Table 3:** The prediction results of 61 three-finger toxins.

	Adaptive Base Classifier				Two-Step Method			
	TP	FP	FN	TN	TP	FP	FN	TN
Cytolysis	7	3	0	51	6	1	1	53
Cardiotoxicity	7	0	0	54	7	0	0	54
Neurotoxicity	31	19	0	11	30	18	1	12
Presynaptic	0	0	0	61	0	0	0	61
Postsynaptic	22	7	8	24	22	7	8	24
Lipid binding	0	0	0	61	0	0	0	61
Vasoactivity	0	0	0	61	0	0	0	61
Hypotension	0	0	0	61	0	1	0	60
Hemolysis	0	0	2	59	0	0	2	59
Hemostasis	0	2	0	59	0	1	0	60

## Data Availability

Data are contained within the article or Supplementary Materials.

## Supplementary Materials

The following supporting information can be downloaded at: https://www.mdpi.com/article/10.3390/toxins14110811/s1, List S1: Physicochemical descriptors; Table S1. The top models from adapting base classifier and their evaluation metrics for the test set of each activity category; Table S2. The evaluation metrics of the top hemolysis models from adapting base classifier on HemoPI dataset after removing the outliers; Table S3. The evaluation metrics of the top models from adapting base classifier on the test set after feature selection; Table S4. The evaluation metrics and the optimal hyperparameters of the top models from adapting base classifier; Table S5. The top models from the two-step method and their evaluation metrics for the test set of each activity category; Table S6. The evaluation metrics of the top models from the two-step method on the test set after feature selection; Table S7. The evaluation metrics and the optimal hyperparameters of the top models from the two-step method; Table S8. The top models from PU bagging and their evaluation metrics for the test set of each activity category; Table S9. The information of 61 three-finger toxins; Table S10. The prediction results of 61 three-finger toxins using top models based on adaptive base classifier and two-step method.
