# Supplementary material for: Function Prediction of Peptide Toxins with Sequence-Based Multi-Tasking PU Learning Method
Source: Toxins (Basel). 2022 Nov 21;14(11):811. doi: 10.3390/toxins14110811 (PMC9696491; doi:10.3390/toxins14110811)
Supplement: Supplementary file 1 [file toxins-14-00811-s001.zip › toxins-1911085-supplementary.pdf]

Supplementary Materials: Functional prediction of peptide toxins with sequence-based multi-task PU learning method

Yanyan Chu, Huanhuan Zhang and Lei Zhang

List S1. Physicochemical descriptors.
Implementation using Python modules and references for all descriptors (https://modlamp.org/modlamp.html#module-modlamp.descriptors)
InstabilityIndex: protein stability based on the amino acid composition;
Length: peptide length;
MW: molecular weight;
NetCharge: overall charge;
IsoelectricPoint: isoelectric point;
Aromaticity: aromaticity, it is simply the relative frequency of Phe+Trp+Tyr;
AliphaticIndex: a measure of thermal stability of proteins and is dependant on the relative volume occupied by aliphatic amino acids;
BomanIndex: a measure for protein-protein interactions;
HydrophobicRatio: the relative frequency of the amino acids A, C, F, I, L, M and V;
S_AASI/ uS_AASI: amino acid selectivity index scale for helical antimicrobial peptides index;
modlabs_ABHPRK: modlabs inhouse physicochemical feature scale (Acidic, Basic, Hydrophobic, Polar, aRomatic, Kink-inducer);
H_argos/ uH_argos: hydrophobicity index with Argos hydrophobicity amino acid scale;
B_Bulkiness/ uB_Bulkiness: Amino acid side chain bulkiness scale;
charge_phys: amino acid charge at pH 7.0 - Hystidine charge +0.1;
charge_acid: amino acid charge at acidic pH - Hystidine charge +1.0;
H_Eisenberg/ uH_Eisenberg: Eisenberg hydrophobicity consensus amino acid scale;
Ez: potential that assesses energies of insertion of amino acid side chains into lipid bilayers;
flexibility/ u_flexibility: amino acid side chain flexibility scale;
Grantham: amino acid side chain composition, polarity and molecular volume;
H_GRAVY/ uH_GRAVY: GRAVY hydrophobicity amino acid scale;
H_HoppWoods/ uH_HoppWoods: Hopp-Woods amino acid hydrophobicity scale;
ISAECI: Isotropic Surface Area (ISA) and Electronic Charge Index (ECI) of amino acid side chains;
H_Janin/ uH_Janin: Janin hydrophobicity amino acid scale;
H_KyteDoolittle/ uH_KyteDoolittle: Kyte & Doolittle hydrophobicity amino acid scale;
F_Levitt/ uF_Levitt: Levitt amino acid a-helix propensity scale;
MSS_shape/ u_MSS_shape: a graph-theoretical index that reflects topological shape and size of amino acid side chains;
MSW: amino acid scale based on a PCA of the molecular surface based WHIM descriptor (MS-WHIM), extended to natural amino acids;
pepArc: modlabs pharmacophoric feature scale, dimensions are: hydrophobicity, polarity, positive charge, negative charge, proline;
pepcats: modlabs pharmacophoric feature based PEPCATS scale;
polarity/ u_polarity: Amino acid polarity scale;
PPCALI: modlabs inhouse scale derived from a PCA of 143 amino acid property scales;
Refractivity/ u_refractivity: Relative amino acid refractivity values;
t_scale: A PCA derived scale based on amino acid side chain properties calculated with 6 different probes of the GRID program;
TM_tend/ u_TM_tend: Amino acid transmembrane propensity scale;
Z3_1, Z3_2, Z3_3: original three dimensional Z-scale;
Z5_1, Z5_2, Z5_3, Z5_4, Z5_5: extended five dimensional Z-scale.

Table S1. The top models from adapting base classifier and their evaluation metrics for the test set of each activity category.

Activity	Model	TPR	AUPR	R	bAcc	F1	P	MCC	AUC-ROC
Cardiotoxicity[a]	KNN	0.76	0.81	1.00	0.88	0.78	1.00	0.78	0.95
Neurotoxicity[a]	LGBM	0.87	0.89	0.84	0.85	0.80	0.85	0.68	0.93
Postsynaptic[a]	KNN	0.71	0.91	0.98	0.85	0.82	0.98	0.82	0.97
Presynaptic[a]	LGBM	0.49	0.68	0.98	0.75	0.63	0.98	0.64	0.96
Cytolysis[a]	LGBM	0.63	0.87	0.99	0.82	0.75	0.99	0.76	0.97
Hemostasis[a]	KNN	0.58	0.58	0.98	0.78	0.57	0.98	0.56	0.88
Hypotension[a]	KNN	0.86	0.84	1.00	0.93	0.84	1.00	0.84	0.98
Lipid binding[a]	LGBM	0.89	0.89	1.00	0.94	0.89	1.00	0.89	1.00
Vasoactivity[a]	KNN	0.87	0.90	1.00	0.93	0.90	1.00	0.90	1.00
Hemolysis[a]	LGBM	0.59	0.72	0.99	0.80	0.72	0.99	0.73	0.96
HemoPI1[a]		0.28	0.71	0.62	0.62	0.42	0.73	0.33	0.63
HemoPI2[a]		0.28	0.66	0.55	0.58	0.40	0.62	0.19	0.58
HemoPI3[a]		0.24	0.64	0.53	0.56	0.36	0.60	0.14	0.57
Hemolysis[b]	LGBM	0.78	0.85	0.97	0.88	0.80	0.97	0.78	0.98
HemoPI1[b]		0.98	0.98	0.94	0.94	0.94	0.94	0.88	0.98
HemoPI3[b]		0.81	0.88	0.80	0.80	0.82	0.80	0.60	0.85

Note: [a] represents the model was trained based on UniprotKB;
[b] represents the model was trained based on UniprotKB and HemoPI2 combined dataset.

Table S2. The evaluation metrics of the top hemolysis models from adapting base classifier on HemoPI dataset after removing the outliers.

Data Set	Model	TPR	bAcc	P	R	MCC	F1	AUC-ROC	AUPR
HemoPI1	LGBM	0.8	0.9	0.92	0.9	0.82	0.89	0.84	0.91
HemoPI2	LGBM	0.68	0.76	0.77	0.77	0.53	0.8	0.84	0.9
HemoPI3	LGBM	0.68	0.73	0.73	0.73	0.46	0.72	0.8	0.86

Table S3. The evaluation metrics of the top models from adapting base classifier on the test set after feature selection.

Activity	Model	TPR	AUPR	R	bAcc	F1	P	MCC	AUC-ROC
**Cardiotoxicity[a]**	KNN	0.76	0.82	1.00	0.88	0.78	1.00	0.78	0.95
Neurotoxicity[a]	LGBM	0.85	0.89	0.84	0.84	0.79	0.85	0.66	0.93
Postsynaptic[a]	LGBM	0.71	0.92	0.98	0.86	0.83	0.98	0.83	0.97
Presynaptic[a]	LGBM	0.57	0.69	0.98	0.78	0.67	0.98	0.67	0.96
Cytolysis[a]	LGBM	0.66	0.86	0.99	0.83	0.78	0.99	0.78	0.97
Hemostasis[a]	KNN	0.51	0.54	0.98	0.75	0.53	0.98	0.52	0.88
Hypotension[a]	KNN	0.95	0.85	1.00	0.97	0.83	1.00	0.84	0.98
Lipid binding[a]	LGBM	0.89	0.92	1.00	0.94	0.91	1.00	0.91	1.00
Vasoactivity[a]	KNN	0.87	0.93	1.00	0.93	0.93	1.00	0.93	1.00
Hemolysis[b]	LGBM	0.77	0.85	0.97	0.88	0.79	0.97	0.77	0.98
HemoPI1[b]		0.98	0.98	0.93	0.93	0.94	0.94	0.87	0.98
HemoPI3[b]		0.81	0.88	0.81	0.81	0.82	0.81	0.61	0.85

Note: [a] represents the model was trained based on UniprotKB;
[b] represents the model was trained based on UniprotKB and HemoPI2 combined dataset.

Table S4. The evaluation metrics and the optimal hyperparameters of the top models from adapting base classifier.

Activity	Model	Parameters	TPR	AUPR	R	bAcc	F1	P	MCC	AUC-ROC
Cardiotoxicity[a]	KNN	{'n_neighbors': 5, 'p': 1, 'weights': 'distance'}	0.81	0.83	0.81	0.90	0.83	0.85	0.83	0.95
Neurotoxicitya	LGBM	{'max_depth': 8, 'num_leaves': 40}	0.89	0.90	0.86	0.81	0.74	0.85	0.70	0.94
Postsynaptica	LGBM	{'max_depth': 8, 'num_leaves': 40}	0.91	0.82	0.91	0.86	0.90	0.98	0.85	0.97
Presynaptica	LGBM	{'max_depth': 8, 'num_leaves': 40}	0.70	0.66	0.71	0.85	0.65	0.61	0.64	0.95
Cytolysisa	LGBM	{'max_depth': 7, 'num_leaves': 30}	0.80	0.87	0.80	0.90	0.85	0.90	0.84	0.97
Hemostasis[a]	KNN	{'n_neighbors': 3, 'p': 4, 'weights': 'distance'}	0.60	0.58	0.60	0.80	0.61	0.63	0.60	0.88
Hypotension[a]	KNN	{'n_neighbors': 6, 'p': 1, 'weights': 'distance'}	0.86	0.89	0.86	0.93	0.88	0.90	0.88	0.97
Lipid binding[a]	LGBM	{'max_depth': 8, 'num_leaves': 20}	0.94	0.87	0.94	0.97	0.85	0.77	0.85	0.99
Vasoactivity[a]	KNN	{'n_neighbors': 5, 'p': 2, 'weights': 'distance'}	0.93	0.99	0.93	0.97	0.93	0.93	0.93	1.00
Hemolysis[b]	LGBM	{'max_depth': 7, 'num_leaves': 20}	0.82	0.84	0.82	0.90	0.78	0.74	0.76	0.98
HemoPI1[b]		{'max_depth': 7, 'num_leaves': 20}	0.99	0.96	0.99	0.85	0.87	0.78	0.73	0.95
HemoPI3[b]		{'max_depth': 7, 'num_leaves': 20}	0.87	0.88	0.87	0.78	0.82	0.77	0.57	0.84

Note: [a] represents the model was trained based on UniprotKB;
[b] represents the model was trained based on UniprotKB and HemoPI2 combined dataset.

Table S5. The top models from the two-step method and their evaluation metrics for the test set of each activity category.

Activity	Model2	TPR	AUPR	R	bAcc	F1	P	MCC	AUC-ROC
Cardiotoxicity[a]	KNN	0.76	0.80	1.00	0.88	0.80	1.00	0.80	0.95
Neurotoxicity[a]	LGBM	0.78	0.89	0.85	0.83	0.78	0.85	0.67	0.93
Postsynaptic[a]	LGBM	0.69	0.91	0.97	0.84	0.81	0.97	0.81	0.97
Presynaptic[a]	LGBM	0.34	0.62	0.98	0.67	0.49	0.98	0.54	0.94
Cytolysis[a]	LGBM	0.60	0.86	0.99	0.80	0.74	0.98	0.75	0.97
Hemostasis[a]	KNN	0.33	0.50	0.98	0.66	0.44	0.98	0.46	0.88
Hypotension[a]	KNN	0.86	0.90	1.00	0.93	0.90	1.00	0.90	0.98
Lipid binding[a]	SVC_poly	0.83	0.86	1.00	0.92	0.86	1.00	0.86	0.94
Vasoactivity[a]	KNN	0.80	0.90	1.00	0.90	0.89	1.00	0.89	1.00
Hemolysis[a]	LGBM	0.57	0.73	0.99	0.79	0.70	0.99	0.71	0.96
HemoPI1[a]		0.23	0.69	0.60	0.60	0.36	0.73	0.30	0.62
HemoPI2[a]		0.23	0.65	0.54	0.57	0.35	0.64	0.19	0.57

HemoPI3[a]		0.19	0.62	0.52	0.55	0.31	0.61	0.14	0.56
Hemolysis[b]	LGBM	0.75	0.84	0.97	0.87	0.78	0.97	0.76	0.98
HemoPI1[b]		0.98	0.99	0.95	0.95	0.95	0.95	0.90	0.99
HemoPI3[b]		0.79	0.85	0.79	0.79	0.81	0.79	0.59	0.85

Note: [a] represents the model was trained based on UniprotKB;
[b] represents the model was trained based on UniprotKB and HemoPI2 combined dataset.

Table S6. The evaluation metrics of the top models from the two-step method on the test set after feature selection.

Activity	Model2	TPR	AUPR	R	bAcc	F1	P	MCC	AUC-ROC
Cardiotoxicity[a]	KNN	0.76	0.82	1.00	0.88	0.80	1.00	0.80	0.95
Neurotoxicity[a]	LGBM	0.80	0.88	0.85	0.84	0.79	0.85	0.68	0.93
Postsynaptic[a]	LGBM	0.68	0.81	0.96	0.83	0.75	0.96	0.73	0.95
Presynaptica	LGBM	0.33	0.63	0.98	0.66	0.48	0.97	0.53	0.94
Cytolysis[a]	LGBM	0.62	0.87	0.99	0.81	0.76	0.99	0.77	0.97
Hemostasis[a]	KNN	0.33	0.52	0.98	0.66	0.44	0.98	0.46	0.88
Hypotension[a]	KNN	0.81	0.86	1.00	0.90	0.85	1.00	0.85	0.98
Lipid binding[a]	SVC_poly	0.67	0.91	1.00	0.83	0.80	1.00	0.82	0.99
Vasoactivity[a]	KNN	0.80	0.98	1.00	0.90	0.89	1.00	0.89	1.00
Hemolysis[b]	LGBM	0.76	0.85	0.97	0.87	0.80	0.97	0.78	0.98
HemoPI1[b]		0.97	0.99	0.94	0.94	0.95	0.95	0.89	0.99
HemoPI3[b]		0.77	0.84	0.79	0.80	0.80	0.80	0.59	0.85

Note: [a] represents the model was trained based on UniprotKB;
[b] represents the model was trained based on UniprotKB and HemoPI2 combined dataset.

Table S7. The evaluation metrics and the optimal hyperparameters of the top models from the two-step method.

Activity	Model2	Parameters	TPR	AUPR	R	bAcc	F1	P	MCC	AUC-ROC
Cardiotoxicity[a]	KNN	{'n_neighbors': 5, 'p': 1, 'weights': 'distance'}	0.81	0.87	1.00	0.90	0.85	1.00	0.85	0.95
Neurotoxicity[a]	LGBM	{'max_depth': 8, 'num_leaves': 40}	0.85	0.90	0.86	0.86	0.81	0.86	0.70	0.94
Postsynaptic[a]	LGBM	{'max_depth': 8, 'num_leaves': 40}	0.80	0.91	0.98	0.90	0.86	0.98	0.86	0.97
Presynaptic[a]	LGBM	{'max_depth': 8, 'num_leaves': 40}	0.51	0.64	0.98	0.75	0.57	0.97	0.56	0.94
Cytolysis[a]	LGBM	{'max_depth': 7, 'num_leaves': 30}	0.77	0.87	0.99	0.88	0.81	0.99	0.80	0.97
Hemostasis[a]	KNN	{'n_neighbors': 3, 'p': 4, 'weights': 'distance'}	0.36	0.55	0.98	0.68	0.48	0.98	0.50	0.87
Hypotension[a]	KNN	{'n_neighbors': 6, 'p': 1, 'weights': 'distance'}	0.86	0.86	1.00	0.93	0.86	1.00	0.86	0.93
Lipid binding[a]	SVC_poly	{'max_depth': 8, 'num_leaves': 20}	0.94	0.92	1.00	0.97	0.90	1.00	0.90	0.99
Vasoactivity[a]	KNN	{'n_neighbors': 5, 'p': 2, 'weights': 'distance'}	0.87	1.00	1.00	0.93	0.93	1.00	0.93	1.00

Hemolysis[b]	LGBM	{'max_depth': 7, 'num_leaves': 20}	0.82	0.85	0.96	0.90	0.78	0.97	0.76	0.98
HemoPI1[b]		{'max_depth': 7, 'num_leaves': 20}	0.99	0.98	0.87	0.87	0.88	0.89	0.75	0.98
HemoPI3[b]		{'max_depth': 7, 'num_leaves': 20}	0.85	0.85	0.79	0.78	0.81	0.79	0.57	0.85

Note: [a] represents the model was trained based on UniprotKB;
[b] represents the model was trained based on UniprotKB and HemoPI2 combined dataset.

Table S8. The top models from PU bagging and their evaluation metrics for the test set of each activity category.

Activity	Model	TPR	AUPR	R	bAcc	F1	P	MCC	AUC-ROC
Neurotoxicity	LGBM	0.87	0.82	0.82	0.83	0.78	0.84	0.64	0.91
Cytolysis	QDA	0.81	0.74	0.94	0.88	0.52	0.97	0.53	0.96
Lipid binding	ADA	0.89	0.78	0.94	0.92	0.23	0.99	0.33	0.97
Vasoactivity	GB	0.95	0.58	0.94	0.94	0.23	0.99	0.34	0.94
Postsynap	QDA	0.89	0.80	0.91	0.90	0.63	0.95	0.62	0.96
Presynap	DTC	0.83	0.41	0.85	0.84	0.27	0.97	0.32	0.90
Hemolysis	QDA	0.75	0.77	0.98	0.87	0.65	0.98	0.65	0.97
Hemostasis	DTC	0.89	0.34	0.83	0.86	0.20	0.98	0.28	0.91
Cardiotoxicity	RF	0.96	0.79	0.96	0.96	0.38	0.99	0.46	0.96
Hypotension	GB	0.92	0.78	0.92	0.92	0.22	0.99	0.32	0.98

Table S9. The information of 61 three-finger toxins.

UniprotID	Name	Sequence
U3EPL2	3FTx 3b	LKCYSSRTETMTCPEGEDKCEKYAAGLMHGSFFFIYTCTSKCHEGAYNVCCST DLCNKSSTSG
F5CPD3	3FTx MALT0044C	RICDDSNIPSERTPKRCQGGYNICYKINFPTPGYELLQIKGCAARCPTNPRFPKA ECCA
F5CPE3	3FTx MALT0065C	VTCHKTDVFTKTCISPICYEKITSAFIIERGCGCPETSRKVKVRCCMTDKCNR
C6JUP1	3FTx-1	LICYNTMMQKVTCPEGKDKCEKYAVPVMRGKFYFSYQCTSKCHEGAYDVCC STDLCNKS
C6JUP2	3FTx-2	ANTLFCDNSNVPSIRTRKRCLKNQKLCYKMTFFTPGFGWTQIKGCIHRCPESTP NEKYQCCSTDNCI
A7X4T2	3FTx-Oxy6	LKCHESENLDDHVVCEEDETMCYKFTFVPFRDFEIVARGCSASCPEEKDVVCC STDLCN
A2CKF6	3FTx-VIIIa	LTCLICPERYCQKVHTCRGEEKLCVKRFYDEKALGWRAKRGCAATCPEAKPK ETVECCSTDKCNK
K9MCX0	A2	LVCYVSGAWQKTCPEGQNKCEKYAVGTMHGSWIYLRGCASTCHEGPYNVC CSTDLCNK
D2N118	a-bungarotoxin	YTIVCHTTATSPISAVTCPPGENLCYRKMWCDAFCSSRGKVVELGCAATCPSK KPYEEVTCCSTDKCNPHPKQRPG
P01380	a-EPTX-Ast2a	LSCYLGYKHSQTCPPGENVCFVKTWCDGFCNTRGERIIMGCAATCPTAKSGV HIACCSTDNCNIYAKWGS
P01381	a-EPTX-Ast2b	LSCYLGYKHSQTCPPGENVCFVKTWCDAFCSTRGERIVMGCAATCPTAKSGV HIACCSTDNCNIYTKWGSGR
P60770	Cobrotoxin (CBT)	LECHNQQSSQTPTTTGCSGGETNCYKKRWRDHRGYRTERGCGCPSVKNGIEI NCCTTDRCNN
P80958	Cobrotoxin-b	LECHNQQSSQTPTTKTCSGETNCYKKWWSDHRGTIIERGCGCPKVKPGVNLN CCTTDRCNN

P62375	CTX A5	LKCHNTQLPFIYKTCPEGKNLCFKATLKKFPLKFPVKRGCADNCPKNSALLKYVCCSTD
P60305	CTX1	LKCNKLIPIASKTCPAGKNLCYKMFMMSDLTIPVKRGCIDVCPKNSLLVKYVCCNTDRCN
P80245	CTX6	LKCNQLIPPFYKTCAAGKNLCYKMFMVAAPKVPVKRGCIDVCPKSSLLVKYVCCNTDRC
Q98965	CTX6)	LKCNQLIPPFYKTCAAGKNLCYKMFMVAAQRFPVKRGCIDVCPKSSLLVKYVCCNTDRC
P49122	CTX7	LKCHNTQLPFIYNTCPEGKNLCFKATLKFPLKFPVKRGCAATCPRSSSLVKVVCCKTDK
P49123	CTX8	LKCNQLIPPFYKACAAGKNLCYKMFMVAAPKVPVKRGCIDVCPKSSLLVKYVCCNTDRC
Q91137	Cytotoxin homolog 5	LKCHNTQLPFIYKTCPEGKNLCFKATLKKFPLKFPVKRGCADNCPKNSALLKYVCCSSD
Q91136	Cytotoxin I-like T-15	LKCNKLIPIASKTCTAGKNLCYKMFMMSDLTIPVKRGCIDVCPKNSLLVKYVCCNTDRC
P60303	Cytotoxin IV	LKCNKLVPLFYKTCPAGKNLCYKMFMVATPKVPVKRGCIDVCPKSSLLVKYVCCNTDRC
U3FAE1	Fulditoxin	LKCYSSRTETMTCPEGEDKCEKYAVGLMHGSFFFIYTCTSKCHEGAYNVCCSTDLCNKSSTSG
P25494	Hydrophitoxin a	MTCCNQQSSQPKTTTNCAESSCYKKTWSDHRGTRIERGCGCPQVKKGIKLECCHTNECNN
P62376	Hydrophitoxin b	MTCCNQQSSQPKTTTNCAESSCYKKTWSDHRGTRIERGCGCPQVKSGIKLECCHTNECNN
U3F5B1	Long chain neurotoxin 4	TRKSVTCPKGEKVCYTIFLVGPSYPEKVLKWGCAASCPKVGLGARISCCSR
F5CPD5	MALT0051C)	MICYNQQSSQPPTTTTCSEGQCYKKTWSDHRGTIIERGCACPNVKPGVKISCCSSDKCNG
F5CPD6	MALT0052C)	LICYNDHGYIGKTTETCENGMTTCYEERWREARGTRIERGCGCYKVKPGVQMNCCKTDRCNG
F5CPD7	MALT0054C)	LICYNYWTPLDKTTECCGNGVTTCFAKSWNDHRGRRTDRGCGCPNVKPGIHLNCCKTDRCNG
F5CPD8	MALT0057C	MICYNQQSSQPPTTTTCSEGQCYKQRWRDHRGWRTERGCGCPKAIPEVKLNCCKTDRCNG
F5CPD9	MALT0058C	LICYNDHGFIGKTTETCENGMTTCYEKRWTEARGTRIDRGCGCPNVKPGVNLNCCKTDRCNG
F5CPE0	MALT0059C	LICYVTRDGKTATCPPGQKCEKYAVSASHTGHWFHRWHCTSTCHEGPYNVCCSTDFCNR
F5CPE2	MALT0063C	LICYVSEYGAKMTCPEGKTLCEKYAVPLMQGHFYFAWRCTSTCKAGAYNICCSTDLCNK
F5CPE4	MALT0066C	LICYNYETPLDKTTECCGNGVTTCFAKSWRDHRGLRTDRGCGCPNVKPGVTINCCKTDRCNG
F5CPE6	MALT0070C	LKCYVGRKPYKLITCPEGSKKCATVPLPTRPLPIFSKGCYTSCPSQYVKCCSTDLCNGS
C6JUP3	MicTx3	LVCYTNVLEPPGTLETCPDDFTCVKKWEGGGRRVTQYCSHACAIPASYEFVHCCQTDKC
K9MCH1	MlatA1	RICYNQQSSQPPTTKTCSEGQCYKKTWRDHRGTIIERGCACPNVKPGIQISCCTSDKCNG
P80548	Mnn I	MICHNQQSSQPPTIKTCSEGQCYKKTWRDHRGTISERGCGCPTVKPGIHISCCASDKCNA
P86095	MS1	MICYNQQSTEPPTTKTCSEGQCYKKTWSDHRGTIIERGCACPNVKPGVKISCCSSDKCR

P86097	MS3	LICYSQMYNEIIKTCENGETTCYSKTWRDHRGTRLEKGCGCPPVKYDMIVKCCKTDRCGN
Q9PSN6	Neurotoxin 3	LECHDQQSSQTPTTTGCSGGETNCYKKRWRDHRGYRTERGCGCPSVKNGIEINCCTTDRCNN
D5J9P6	Non-conventional three finger toxin isoform 2	KCKICQFNTCRPGELKVCSGEEIYCFKESWSTARGTRIERGCTATCPKGSVYGNYVLC
D5J9P8	Non-conventional three finger toxin isoform 4	YTIKCKICQFNTCRPGELKVCSGEEIYCFKESWSTARGTRIERGCTATCPKGSVYGNYVLC
D5J9P9	Non-conventional three finger toxin isoform 5	CLICPEKYCQKVHTCQDAEKICFKRFYEGKQLGKKFPRGCAATCPEAKPHEIVECCS
D5J9Q0	Non-conventional three finger toxin isoform 6	CLICPEKYCQKVHTCRDGEKICFKRFYEGKRFGKKFPRGCAATCPEAKPHEIVECCS
P60774	NTX I	LECHNQQSSQAPTTKTCSGETNCYKKWWSDHRGTIIERGCGCPKVKPGVKLNCCTTDRCNN
Q6IZ95	Ntx4	LTCLICPEKYCQKVHTCRDGENLCVKRFYEGKRFGKKYPRGCAATCPEAKPHEIVECCSTDKCNK
D5J9N9	Short-chain three finger toxin isoform 2	NVCYTHESANPKTSVLCGYGTIFCYKSSWIYRGVEKIERGCASACPDMKPNGKYIYCCT
D5J9P0	Short-chain three finger toxin isoform 3	NVCYTHESANPKTSVLCGYGTIFCYKSSWIYRGVEKIERGCASACPDMKPNGKHIYCCT
D5J9P1	Short-chain three finger toxin isoform 4	RICYNQQSTTPPTTENCEPGKNVCYKMYFSDHRGTRSSRGCVATCPTNNRYDRVVCCEK
D5J9P2	Short-chain three finger toxin isoform 5	RICYNQQSTTPPTTENCEPGKNVCYKMYFSDHRGTRSSRGCVVTCPTNNRYDRVVCCEK
D5J9P3	Short-chain three finger toxin isoform 6	KLCYNHQSTNPKTTELCGHSMYFCYKNSWIYRGVEKIERGCSLTCPDIKSNGKYIYCCT
D5J9P4	Short-chain three finger toxin isoform 7	RKCLTKYSRDNESSKTCPSGQNVCFKKWEMGNSSEKNAKRGCIAACPKPKKNEMIQCCS
E2IU15	Three-finger toxin	MKCKDMSLRYFVVPVNSKFVQVERSIAFKESWREARGTRIERGCAATCPKGSVYGLYVL
A5X2W8	Three-finger toxin 3	EPGYTTNCFTCTTWTLSCREFEKCPPDKGTCFKRWNSTGIAIRRRYTRGCAAACPNPVGNEKVF
P25668	Toxin A	IRCFITPDITSKDCPNGHVCYTKTWCDGFCSIRGKRVDLGCAATCPTVRTGVDIQCCSTDDCDPFPTRKRP
P0CG02	Toxin aagardi	MTCCNQQSSQPKTTTNCAGNSCYKKTWSDHRGTIIERGCGCPQVKSGIKLECCHTNECNN
C0HLK3	Tschuditoxin-I	MICYNQQSSEPPTTKTCSEGQCYKKTWSDHRGTIIERGCACPNVKPGVKISCCSSDK
C1IC47	W-III	FVCHNQQSSQPPTTTNCSGGENKCYKKQWSDHRGSITERGCGCPTVKKGIKLHCCTTEKCNN
C1IC48	W-IV	LLCHNQQSSTSPTTTCCSGGESKCYKKRWPTHRGTITERGCGCPTVKKGIELHCCTTDQCNL
C1IC49	W-V	LTCLICPKKYCNQVHTCRNGENLCIKTFYEGNLLGKQFKRGCAATCPEARPREIVECCSRDKCNH

Table S10. The prediction results of 61 three-finger toxins using top models based on adaptive base classifier and two-step method.

Name	UniprotID	Cardiotoxicity	Neurotoxicity	Presynaptic	Postsynaptic	Lipid binding	Vasoactivity	Hypotension	Cytolysis	Hemolysis	Hemostasis
3FTx 3b	U3EPL2	-/-/-	+/+/-	-/-/-	+/-/-	-/-/-	-/-/-	-/-/-	-/-/-	-/-/-	-/-/-
3FTx MALT0044C	F5CPD3	-/-/-	-/+/-	-/-/-	-/+/+	-/-/-	-/-/-	-/-/-	-/-/-	-/-/-	-/-/-

3FTx MALT0065C	F5CPE3	-/-/-	-/+/+	-/-/-	-/-/-	-/-/-	-/-/-	-/-/-	-/-/-	-/-/-	-/-/-
3FTx-1	C6JUP1	-/-/-	-/+/+	-/-/-	-/-/-	-/-/-	-/-/-	-/-/-	-/-/-	-/-/-	-/-/-
3FTx-2	C6JUP2	-/-/-	+/+/+	-/-/-	+/+/+	-/-/-	-/-/-	-/-/-	-/-/-	-/-/-	-/-/-
3FTx-Oxy6	A7X4T2	-/-/-	-/+/+	-/-/-	-/-/-	-/-/-	-/-/-	-/-/-	-/-/-	-/-/-	-/-/-
3FTx-VIIIa	A2CKF6	-/-/-	+/+/+	-/-/-	+/+/+	-/-/-	-/-/-	-/-/-	-/-/-	-/-/-	-/-/-
3FTx A2	K9MCX0	-/-/-	-/+/+	-/-/-	-/-/-	-/-/-	-/-/-	-/-/-	-/-/-	-/-/-	-/+/-
α-bungarotoxin	D2N118	-/-/-	-/+/+	-/-/-	-/-/-	-/-/-	-/-/-	-/-/-	-/-/-	-/-/-	-/-/-
α-EPTX-Ast2a	P01380	-/-/-	+/+/+	-/-/-	+/-/-	-/-/-	-/-/-	-/-/-	-/-/-	-/-/-	-/-/-
α-EPTX-Ast2b	P01381	-/-/-	+/+/+	-/-/-	+/-/-	-/-/-	-/-/-	-/-/-	-/-/-	-/-/-	-/-/-
Cobrotoxin (CBT)	P60770	-/-/-	+/+/+	-/-/-	+/+/+	-/-/-	-/-/-	-/-/-	-/-/-	-/-/-	-/-/-
Cobrotoxin-b	P80958	-/-/-	+/+/+	-/-/-	+/+/+	-/-/-	-/-/-	-/-/-	-/-/-	-/-/-	-/-/-
CTX A5	P62375	-/-/-	-/-/-	-/-/-	-/-/-	-/-/-	-/-/-	-/-/-	-/+/-	-/-/-	-/-/-
CTX1	P60305	+/+/+	-/-/-	-/-/-	-/-/-	-/-/-	-/-/-	-/-/-	+/+/+	-/-/-	-/-/-
CTX6	P80245	+/+/+	-/-/-	-/-/-	-/-/-	-/-/-	-/-/-	-/-/-	+/+/+	+/-/-	-/-/-
CTX6	Q98965	+/+/+	-/-/-	-/-/-	-/-/-	-/-/-	-/-/-	-/-/-	+/+/+	-/-/-	-/-/-
CTX7	P49122	+/+/+	-/-/-	-/-/-	-/-/-	-/-/-	-/-/-	-/-/+	-/+/+	-/-/-	-/-/-
CTX8	P49123	+/+/+	-/-/-	-/-/-	-/-/-	-/-/-	-/-/-	-/-/-	+/+/+	-/-/-	-/-/-
Cytotoxin homolog 5	Q91137	-/-/-	-/-/-	-/-/-	-/-/-	-/-/-	-/-/-	-/-/-	+/+/-	+/-/-	-/-/-
Cytotoxin I-like T-15	Q91136	+/+/+	-/-/-	-/-/-	-/-/-	-/-/-	-/-/-	-/-/-	+/+/+	-/-/-	-/-/-
Cytotoxin IV	P60303	+/+/+	-/-/-	-/-/-	-/-/-	-/-/-	-/-/-	-/-/-	+/+/+	-/-/-	-/-/-
Fulditoxin	U3FAE1	-/-/-	+/+/+	-/-/-	+/-/-	-/-/-	-/-/-	-/-/-	-/-/-	-/-/-	-/-/-
Hydrophitoxin a	P25494	-/-/-	+/+/+	-/-/-	+/+/+	-/-/-	-/-/-	-/-/-	-/-/-	-/-/-	-/-/-
Hydrophitoxin b	P62376	-/-/-	+/+/+	-/-/-	+/+/+	-/-/-	-/-/-	-/-/-	-/-/-	-/-/-	-/-/-
Long chain neurotoxin 4	U3F5B1	-/-/-	+/+/+	-/-/-	-/-/-	-/-/-	-/-/-	-/-/-	-/-/-	-/-/-	-/-/-
MALT0051C	F5CPD5	-/-/-	+/+/+	-/-/-	+/+/+	-/-/-	-/-/-	-/-/-	-/-/-	-/-/-	-/-/-
MALT0052C	F5CPD6	-/-/-	+/+/+	-/-/-	+/-/-	-/-/-	-/-/-	-/-/-	-/-/-	-/-/-	-/-/-
MALT0054C	F5CPD7	-/-/-	+/+/+	-/-/-	+/-/-	-/-/-	-/-/-	-/-/-	-/-/-	-/-/-	-/-/-
MALT0057C	F5CPD8	-/-/-	+/+/+	-/-/-	+/+/+	-/-/-	-/-/-	-/-/-	-/-/-	-/-/-	-/-/-
MALT0058C	F5CPD9	-/-/-	+/+/+	-/-/-	+/-/+	-/-/-	-/-/-	-/-/-	-/-/-	-/-/-	-/-/-
MALT0059C	F5CPE0	-/-/-	+/+/+	-/-/-	+/+/+	-/-/-	-/-/-	-/-/-	-/-/-	-/-/-	-/-/-
MALT0063C	F5CPE2	-/-/-	-/+/+	-/-/-	-/-/-	-/-/-	-/-/-	-/-/-	-/-/-	-/-/-	-/-/-
MALT0066C	F5CPE4	-/-/-	+/+/+	-/-/-	+/-/-	-/-/-	-/-/-	-/-/-	-/-/-	-/-/-	-/+/+
MALT0070C	F5CPE6	-/-/-	-/-/-	-/-/-	-/-/-	-/-/-	-/-/-	-/-/-	-/-/-	-/-/-	-/-/-
MicTx3	C6JUP3	-/-/-	-/+/+	-/-/-	-/+/+	-/-/-	-/-/-	-/-/-	-/-/-	-/-/-	-/-/-
MlatA1	K9MCH1	-/-/-	+/+/+	-/-/-	+/+/+	-/-/-	-/-/-	-/-/-	-/-/-	-/-/-	-/-/-

Mnn I	P80548	-/-/-	+/+/+	-/-/-	+/+/+	-/-/-	-/-/-	-/-/-	-/-/-	-/-/-	-/-/-
MS1	P86095	-/-/-	+/+/+	-/-/-	+/+/+	-/-/-	-/-/-	-/-/-	-/-/-	-/-/-	-/-/-
MS3	P86097	-/-/-	+/+/+	-/-/-	+/+/+	-/-/-	-/-/-	-/-/-	-/-/-	-/-/-	-/-/-
Neurotoxin 3	Q9PSN6	-/-/-	+/+/+	-/-/-	+/+/+	-/-/-	-/-/-	-/-/-	-/-/-	-/-/-	-/-/-
Non-conventional three finger toxin isoform 2	D5J9P6	-/-/-	-/+/+	-/-/-	-/-/-	-/-/-	-/-/-	-/-/-	-/-/-	-/-/-	-/-/-
Non-conventional three finger toxin isoform 4	D5J9P8	-/-/-	-/+/+	-/-/-	-/-/-	-/-/-	-/-/-	-/-/-	-/-/-	-/-/-	-/-/-
Non-conventional three finger toxin isoform 5	D5J9P9	-/-/-	-/+/+	-/-/-	-/+/+	-/-/-	-/-/-	-/-/-	-/-/-	-/-/-	-/-/-
Non-conventional three finger toxin isoform 6	D5J9Q0	-/-/-	-/+/+	-/-/-	-/-/-	-/-/-	-/-/-	-/-/-	-/-/-	-/-/-	-/-/-
NTX I	P60774	-/-/-	+/+/+	-/-/-	+/+/+	-/-/-	-/-/-	-/-/-	-/-/-	-/-/-	-/-/-
Ntx4	Q6IZ95	-/-/-	+/+/+	-/-/-	+/+/+	-/-/-	-/-/-	-/-/-	-/-/-	-/-/-	-/-/-
Short-chain three finger toxin isoform 2	D5J9N9	-/-/-	-/+/+	-/-/-	-/-/-	-/-/-	-/-/-	-/-/-	-/-/-	-/-/-	-/-/-
Short-chain three finger toxin isoform 3	D5J9P0	-/-/-	-/+/+	-/-/-	-/+/+	-/-/-	-/-/-	-/-/-	-/-/-	-/-/-	-/-/-
Short-chain three finger toxin isoform 4	D5J9P1	-/-/-	-/+/+	-/-/-	-/+/+	-/-/-	-/-/-	-/-/-	-/-/-	-/-/-	-/-/-
Short-chain three finger toxin isoform 5	D5J9P2	-/-/-	-/+/+	-/-/-	-/+/+	-/-/-	-/-/-	-/-/-	-/-/-	-/-/-	-/-/-
Short-chain three finger toxin isoform 6	D5J9P3	-/-/-	-/+/+	-/-/-	-/-/-	-/-/-	-/-/-	-/-/-	-/-/-	-/-/-	-/-/-
Short-chain three finger toxin isoform 7	D5J9P4	-/-/-	-/+/+	-/-/-	-/+/+	-/-/-	-/-/-	-/-/-	-/-/-	-/-/-	-/-/-
Three-finger toxin	E2IU15	-/-/-	-/-/-	-/-/-	-/-/-	-/-/-	-/-/-	-/-/-	-/+/-	-/-/-	-/-/-
Three-finger toxin 3	A5X2W8	-/-/-	-/+/+	-/-/-	-/-/-	-/-/-	-/-/-	-/-/-	-/-/-	-/-/-	-/-/-
Toxin A	P25668	-/-/-	+/+/+	-/-/-	+/+/+	-/-/-	-/-/-	-/-/-	-/-/-	-/-/-	-/-/-
Toxin aagardi	P0CG02	-/-/-	+/+/+	-/-/-	+/+/+	-/-/-	-/-/-	-/-/-	-/-/-	-/-/-	-/-/-
Tschuditoxin-I	C0HLK3	-/-/-	+/+/+	-/-/-	+/+/-	-/-/-	-/-/-	-/-/-	-/-/-	-/-/-	-/-/-

W-III	C1IC47	-/-/-	+/+/+	-/-/-	+/+/+	-/-/-	-/-/-	-/-/-	-/-/-	-/-/-	-/-/-
W-IV	C1IC48	-/-/-	+/+/+	-/-/-	+/+/+	-/-/-	-/-/-	-/-/-	-/-/-	-/-/-	-/-/-
W-V	C1IC49	-/-/-	+/+/+	-/-/-	+/+/+	-/-/-	-/-/-	-/-/-	-/-/-	-/-/-	-/-/-

Note: The results were shown as verified activities/adaptive base classifier result/two-step method result.
'+' and '-' indicate active and inactive, respectively.
